# Supplementary material for: Low-level nutrient enrichment during thermal stress delays bleaching and ameliorates calcification in three Hawaiian reef coral species
Source: PeerJ. 2022 Jul 14;10:e13707. doi: 10.7717/peerj.13707 (PMC9288827; doi:10.7717/peerj.13707)
Supplement: Supplemental Information 1 [file peerj-10-13707-s001.docx]

**Supplementary Information:** Han *et al.* 2022. Insert article citation once approved for publication.

**Tables:**

**Table S1.** Colony sample size (n) by species, treatment, and response variable.

|  |  | Treatments | | | |
| --- | --- | --- | --- | --- | --- |
| Species | **Variable** | **A** | **N** | **NH** | **H** |
| *L. scutaria* | Survivorship | 29 | 29 | 20 | 30 |
|  | Partial Mortality/Bleaching | 29 | 29 | 20 | 30 |
|  | DTB | 0 | 0 | 17 | 30 |
|  | Visual Assessment | 29 | 29 | 20 | 30 |
|  | Calcification (≤ 50%) | 29 | 29 | 17 | 30 |
|  | Calcification | 29 | 29 | 20 | 30 |
| *M. capitata* | Survivorship | 23 | 29 | 19 | 29 |
|  | Partial Mortality/Bleaching | 23 | 29 | 19 | 29 |
|  | DTB | 0 | 0 | 17 | 29 |
|  | Visual Assessment | 23 | 29 | 19 | 29 |
|  | Calcification (≤ 50%) | 23 | 29 | 17 | 28 |
|  | Calcification | 23 | 29 | 19 | 29 |
| *P. acuta* | Survivorship | 29 | 29 | 19 | 29 |
|  | Partial Mortality/Bleaching | 29 | 29 | 19 | 29 |
|  | DTB | 0 | 0 | 17 | 27 |
|  | Visual Assessment | 29 | 29 | 19 | 29 |
|  | Calcification (≤ 50%) | 29 | 29 | 16 | 23 |
|  | Calcification | 29 | 29 | 19 | 29 |

**Table S2**. Mean nutrients concentrations (µM) of three reference nutrient sample locations: South Kāne‘ohe Bay, Intake Pipe, and ambient nutrient treatment mesocosms (A)). Samples were taken during the acclimation and experimental heating phase.

| **Location** | **Phosphate** | **N+N** | **Ammonia** |
| --- | --- | --- | --- |
| South Bay | 0.13 ± 0.03 | 0.04 ± 0.01 | 0.06 ± 0.03 |
| Intake Pipe | 0.64 ± 0.42 | 0.31 ± 0.13 | 0.13 ± 0.07 |
| Mesocosms (A) | 0.18 ± 0.02 | 0.14 ± 0.02 | 0.18 ± 0.03 |

**Table S3.** Pairwise comparisons of mean concentrations (µM) of nitrate+nitrite, ammonium, and phosphate between treatment groups by phase.

**
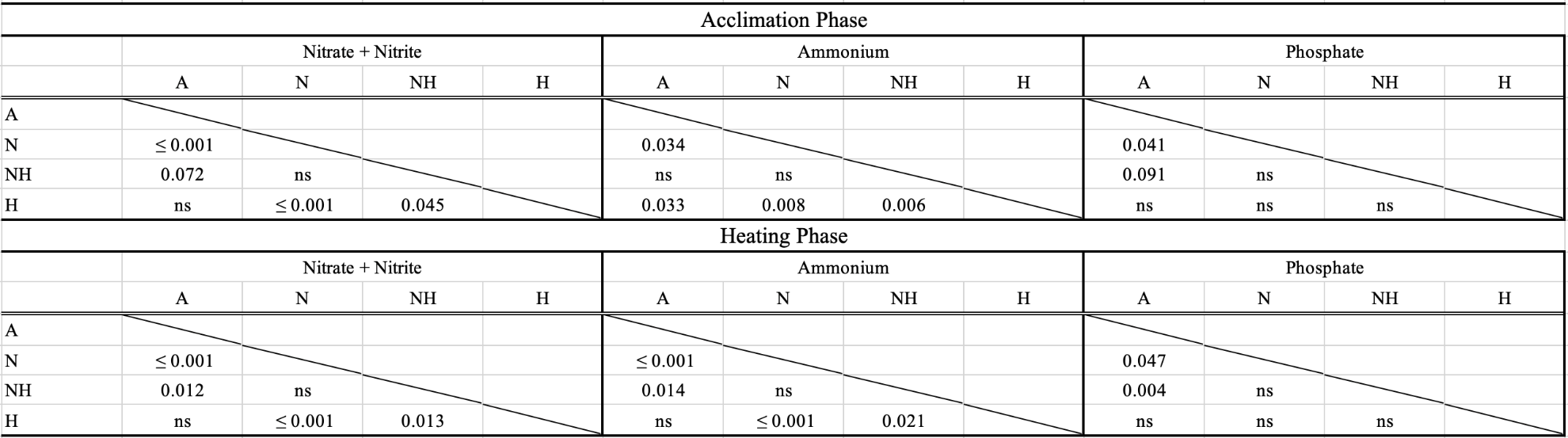
**

**Table S4**. Mean flow rate (sec/10L) with SE for each treatment group.

| **A** | **N** | **NH** | **H** |
| --- | --- | --- | --- |
| 46.82 ± 1.90 | 48.49 ± 0.70 | 48.49 ± 0.70 | 46.61 ± 2.91 |

**Table S5**. Mean peristaltic pump rate (sec/mL) with SE for each treatment group.

| **A** | **N** | **NH** | **H** |
| --- | --- | --- | --- |
| 46.82 ± 1.90 | 48.49 ± 0.70 | 48.49 ± 0.70 | 46.61 ± 2.91 |

**Table S6**. Survivorship (%) of coral colonies by species and treatment groups at the end of the 31 days of the heating phase.

|  | ***L. scutaria*** | ***M. capitata*** | ***P. acuta*** |
| --- | --- | --- | --- |
| A | 100% | 100% | 100% |
| N | 100% | 100% | 100% |
| NH | 85% | 95% | 84% |
| H | 100% | 100% | 86% |

**Table S7.** Mean partial mortality (%) ± SE of coral colonies at the end of the heating phase by species and treatment groups.

|  | ***L. scutaria*** | ***M. capitata*** | ***P. acuta*** |
| --- | --- | --- | --- |
| A | 0 | 0.65 ± 0.65 | 0 |
| N | 0 | 0 | 0 |
| NH | 15.50 ± 8.16 | 17.90 ± 6.24 | 21.30 ± 8.55 |
| H | 0.17 ± 0.17 | 8.62 ± 3.79 | 22.40 ± 7.06 |

**Table S8.** *Post hoc* pairwise comparisons of partial bleaching (%) between treatment groups by species at the end of the heating phase. Bolded *p*-values denote statistically significant differences.

|  | *L. scutaria* | | *M. capitata* | | *P. acuta* | |
| --- | --- | --- | --- | --- | --- | --- |
| Comparison | *z*-ratio | *p-*value | *z*-ratio | *p-*value | *z*-ratio | *p-*value |
| A - H | -13.481 | **≤0.001** | -12.127 | **≤0.001** | -9.577 | **≤0.001** |
| A - N | 0.000 | 1.000 | 0.000 | 1.000 | -0.093 | 1.000 |
| A - NH | -4.002 | **≤0.001** | -7.747 | **≤0.001** | -7.421 | **≤0.001** |
| H - N | 13.481 | **≤0.001** | 12.666 | **≤0.001** | 9.449 | **≤0.001** |
| H - NH | 4.104 | **≤0.001** | 1.301 | 1.000 | 0.406 | 1.000 |
| N - NH | -4.002 | **≤0.001** | -7.932 | **≤0.001** | -7.334 | **≤0.001** |

**Table S9.** Mean ± SE days to bleaching (DTB), calculated as individual colony partial bleaching ≥ 95%, for NH and H treatment groups by species.

|  | ***L. scutaria*** | ***M. capitata*** | ***P. acuta*** |
| --- | --- | --- | --- |
| NH | 20.5 ± 1.3 | 23.3 ± 1.2 | 20.9 ± 0.9 |
| H | 16.4 ± 0.8 | 19.9 ± 0.7 | 18.2 ± 0.7 |

**Table S10.** Mean ± SE days to mortality (DTM), calculated as individual colony partial mortality ≥ 95%, for NH and H treatment groups by species. No mortality was recorded in A and N treatment groups.

|  | ***L. scutaria*** | ***M. capitata*** | ***P. acuta*** |
| --- | --- | --- | --- |
| NH | 40.90 ± 5.75 | 36.80 ± 3.28 | 37.80 ± 2.61 |
| H | 38.70 ± 7.86 | 40.70 ± 3.26 | 37.10 ± 2.66 |

**Table S11.** *Post hoc* pairwise comparisons of mean change in calcification rate (mm/day) between treatment groups by species. Bolded *p*-values denote statistically significant differences.

| Comparison | Estimate | SE | df | *t*-ratio | *p*-value |  |
| --- | --- | --- | --- | --- | --- | --- |
| *L. scutaria* | | | | | | |
| A - H | 0.994 | 0.321 | 874 | 3.102 | **0.012** |  |
| A - N | 0.215 | 0.322 | 862 | 0.667 | 0.505 |  |
| A - NH | 0.642 | 0.353 | 682 | 1.817 | 0.139 |  |
| H - N | 0.779 | 0.321 | 874 | 2.431 | **0.046** |  |
| H - NH | 0.352 | 0.352 | 688 | 1.001 | 0.381 |  |
| N - NH | 0.427 | 0.353 | 682 | 1.208 | 0.341 |  |
| *M. capitata* | | | | | | |
| A - H | 1.362 | 0.336 | 770 | 4.060 | **≤0.001** |  |
| A - N | -0.065 | 0.334 | 780 | -0.194 | 0.846 |  |
| A - NH | 0.779 | 0.364 | 641 | 2.137 | **0.049** |  |
| H - N | 1.427 | 0.324 | 849 | 4.408 | **≤0.001** |  |
| H - NH | 0.584 | 0.355 | 676 | 1.645 | 0.120 |  |
| N - NH | 0.843 | 0.353 | 682 | 2.386 | **0.035** |  |
| *P. acuta* | | | | | | |
| A - H | 2.054 | 0.334 | 780 | 6.150 | **≤0.001** |  |
| A - N | 0.166 | 0.322 | 862 | 0.514 | 0.607 |  |
| A - NH | 0.705 | 0.358 | 664 | 1.970 | 0.074 |  |
| H - N | 1.889 | 0.334 | 780 | 5.654 | **≤0.001** |  |
| H - NH | 1.349 | 0.369 | 626 | 3.660 | **≤0.001** |  |
| N - NH | 0.539 | 0.358 | 664 | 1.507 | 0.159 |  |

**Table S12.** Chi-squared results from Day 12 of heating phase.


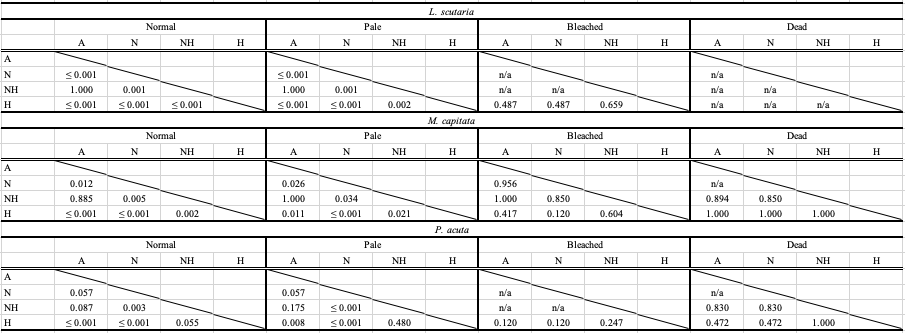


**Table S13.** MCA contribution plots of all variable levels at Day 12.

|  |  | **Dimension 1** |  |  | **Dimension 2** |  |
| --- | --- | --- | --- | --- | --- | --- |
|  | Coordinate | Contribution (%) | cos^2^ | Coordinate | Contribution (%) | cos^2^ |
| A | -0.264 | 1.115 | 0.025 | -0.633 | 9.088 | 0.144 |
| N | -1.105 | 20.180 | 0.460 | 0.344 | 2.776 | 0.045 |
| NH | 0.070 | 0.056 | 0.001 | -0.007 | 0.001 | 0.000 |
| H | 1.297 | 28.145 | 0.644 | 0.269 | 1.718 | 0.028 |
| Ls | 0.086 | 0.153 | 0.004 | -0.922 | 24.763 | 0.437 |
| Mc | 0.046 | 0.042 | 0.001 | 0.496 | 6.900 | 0.119 |
| Pd | -0.133 | 0.356 | 0.009 | 0.453 | 5.863 | 0.102 |
| Normal | -0.804 | 21.728 | 0.811 | 0.074 | 0.265 | 0.007 |
| Pale | 0.945 | 21.046 | 0.571 | -0.504 | 8.490 | 0.162 |
| Bleached | 1.615 | 5.951 | 0.102 | 2.520 | 20.568 | 0.249 |
| Dead | 1.138 | 1.230 | 0.021 | 3.808 | 19.570 | 0.232 |

**Table S14.** Chi-squared results from end of heating phase (Day 31).


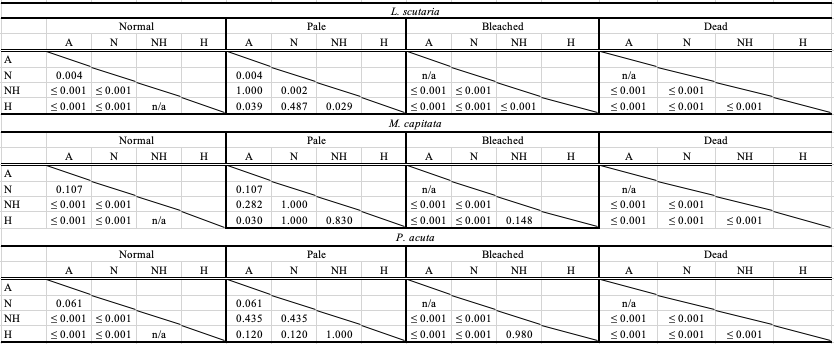


**Table S15.** MCA contribution plots of all variable levels at the end of heating phase (Day 31).

|  |  | **Dimension 1** |  |  | **Dimension 2** |  |
| --- | --- | --- | --- | --- | --- | --- |
|  | Coordinate | Contribution (%) | cos^2^ | Coordinate | Contribution (%) | cos^2^ |
| A | -0.824 | 8.953 | 0.236 | 0.950 | 17.274 | 0.314 |
| N | -1.004 | 14.266 | 0.386 | -0.864 | 15.324 | 0.286 |
| NH | 0.955 | 8.604 | 0.206 | 0.562 | 4.328 | 0.072 |
| H | 1.122 | 18.019 | 0.490 | -0.391 | 3.183 | 0.060 |
| Ls | -0.050 | 0.043 | 0.001 | 0.533 | 7.239 | 0.149 |
| Mc | 0.095 | 0.148 | 0.004 | -0.352 | 2.924 | 0.058 |
| Pd | -0.039 | 0.027 | 0.001 | -0.211 | 1.113 | 0.023 |
| Normal | -0.972 | 22.736 | 0.841 | -0.322 | 3.627 | 0.093 |
| Pale | -0.170 | 0.151 | 0.003 | 2.397 | 43.430 | 0.652 |
| Bleached | 1.120 | 23.077 | 0.705 | -0.237 | 1.503 | 0.032 |
| Dead | 1.079 | 3.977 | 0.083 | -0.106 | 0.055 | 0.001 |

**Figures:**


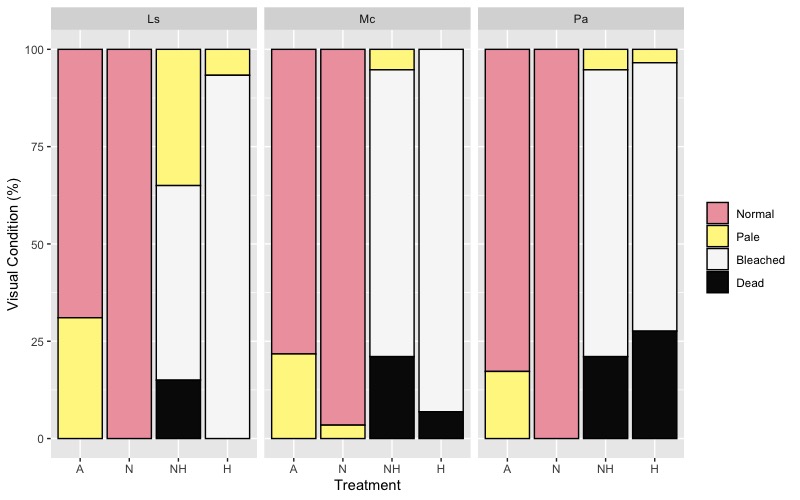


**Figure S1.** Percentage (%) of colonies that were either normal, pale, bleached, or dead by

treatment group and species at the end of the heating period (Day 31).


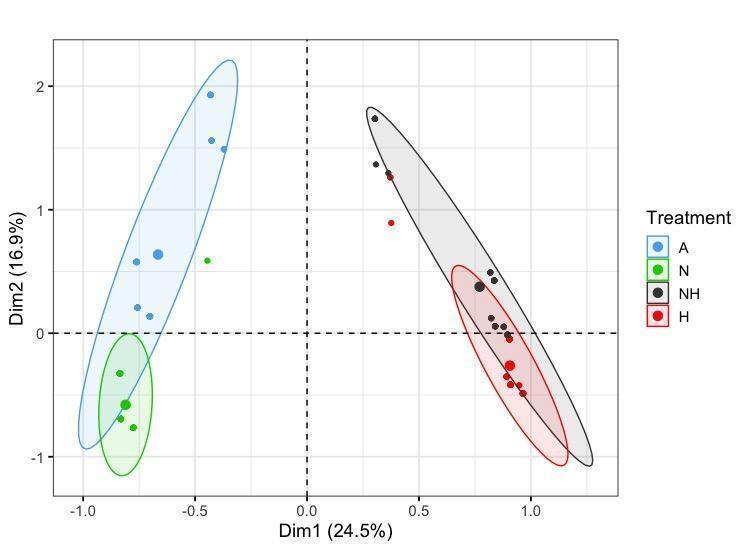


**Figure S2**. MCA plot of treatment, species, and visual assessment data from the end of the

heating period (Day 31). Treatment group centroids are shown along with corresponding 95% CI ellipses.
